# Ca^2+^-Permeable Channels/Ca^2+^ Signaling in the Regulation of Ileal Na^+^/Gln Co-Transport in Mice

**DOI:** 10.3389/fphar.2022.816133

**Published:** 2022-02-23

**Authors:** Fenglan Chu, Hanxing Wan, Weidong Xiao, Hui Dong, Muhan Lü

**Affiliations:** ^1^ Department of Gastroenterology, Affiliated Hospital of Southwest Medical University, Luzhou, China; ^2^ Department of Gastroenterology, Xinqiao Hospital, Army Medical University, Chongqing, China; ^3^ Department of General Surgery, Xinqiao Hospital, Army Medical University, Chongqing, China

**Keywords:** calcium signaling, VGCC, SOCE, CICR, TRPV, NCX

## Abstract

Oral glutamine (Gln) has been widely used in gastrointestinal (GI) clinical practice, but it is unclear if Ca^2+^ regulates intestinal Gln transport, although both of them are essential nutrients for mammals. Chambers were used to determine Gln (25 mM)-induced *I*_*sc*_ through Na^+^/Gln co-transporters in the small intestine in the absence or the presence of selective activators or blockers of ion channels and transporters. Luminal but not serosal application of Gln induced marked intestinal *I*_*sc*_, especially in the distal ileum. Lowering luminal Na^+^ almost abolished the Gln-induced ileal *I*_*sc*_, in which the calcium-sensitive receptor (CaSR) activation were not involved. Ca^2+^ removal from both luminal and serosal sides of the ileum significantly reduced Gln- *I*_*sc*_. Blocking either luminal Ca^2+^ entry *via* the voltage-gated calcium channels (VGCC) or endoplasmic reticulum (ER) release *via* inositol 1,4,5-triphosphate receptor (IP_3_R) and ryanodine receptor (RyR) attenuated the Gln-induced ileal *I*_*sc*_, Likewise, blocking serosal Ca^2+^ entry *via* the store-operated Ca^2+^ entry (SOCE), TRPV1/2 channels, and Na^+^/Ca^2+^ exchangers (NCX) attenuated the Gln-induced ileal *I*_*sc*_. In contrast, activating TRPV1/2 channels enhanced the Gln-induced ileal *I*_*sc*_. We concluded that Ca^2+^ signaling is critical for intestinal Gln transport, and multiple plasma membrane Ca^2+^-permeable channels and transporters play roles in this process. The Ca^2+^ regulation of ileal Na^+^/Gln transport expands our understanding of intestinal nutrient uptake and may be significant in GI health and disease.

## Introduction

Glutamine (Gln) is the most abundant free amino acid (AA) in mammalian plasma and is traditionally considered a non—essential amino acid. Gln is also the most widely used AA in clinical practice, such as in weaning, pregnancy, various gastrointestinal (GI) diseases, as well as the intestinal injury caused by burning (Shi et al., 2018), infection (Liu et al., 2017), and tumor treatment (Takechi et al., 2014) when endogenous Gln is in short supply under some physiological or pathological conditions. Moreover, Gln is regarded as an essential nutritional AA for newborns and a conditional essential AA for adults (Wang et al., 2015). During periods of increased intestinal stress, Gln can stimulate intestinal nutrient absorption while maintaining mucosal barrier function and promoting mucosal growth (Chen et al., 2018; Li et al., 2019). Therefore, investigation on the regulation of Gln uptake will further promote our understanding of its role in GI health and therapeutic application in GI diseases.

Gln transporters can be divided into Na^+^-dependent transporters and Na^+^-independent transporters. Na^+^-dependent transporters expressed differently in different organs, tissues, and cells (Oguchi et al., 2012; Leke et al., 2015; Yamada et al., 2019). Gln is mainly absorbed by Na^+^/Gln co-transporters (NGcT) in the proximal jejunum and ileum. So far, the intestinal NGcT have been classified as follows: system A (SNAT1/2/4), system ASC(ASCT2), system B^0^(B^0^AT1), system B^0,+^ (ATB^0,+^), system N(SNAT3/5/7), and system y^+^L (y^+^LAT1/2). ATB^0,+^and ASCT2 were lowly expressed in the small intestines but high in the large intestines, while the rest were mainly expressed in the small intestine. System A and y^+^L are mainly localized basolaterally in the intestinal epithelium, whereas most of the others are at the apical (Broer and Fairweather 2019). The Na^+^-independent Gln transporters include system asc (Asc-1), b^0,+^ (b^0,+^AT), and L (LAT1/2), in which Gln is transported by exchange with other AA or ions. Although Gln transport pathways, kinetic properties, and energy requirements in different cells and animal models have been extensively investigated, few studies have described the short-term regulation of Gln transport across intestinal epithelial cells (IEC).

As an important second messenger, Ca^2+^ is involved in various physiological processes, such as muscle contraction, hormone secretion, and neurotransmitter release. We have previously reported Ca^2+^ signaling regulation of ion secretion and absorption through SGLT1 in the intestinal epithelium (Zhang F. et al., 2019; Yinghui et al., 2021, Zhang et al., 2021). We also revealed that Ca^2+^ signaling regulates H^+^/peptide transporter PEPT1 to mediate intestinal Gly-Sar uptake in mice (Xu et al., 2020). However, it is currently unknown if Ca^2+^ signaling regulates intestinal Gln uptake.

Given that Ca^2+^ appears to regulate diverse intestinal nutrient uptake, it is reasonable to speculate that Ca^2+^ may also regulate Gln transporters in the gut. Moreover, Gln can enhance intestinal Ca^2+^ absorption, and intracellular Ca^2+^ is involved in the intestinal cell protective effect of Gln (Okuda et al., 2006; Moine et al., 2017), indicating an interaction of intracellular Ca^2+^ and Gln. Therefore, Gln and Ca^2+^ in the intestinal epithelia might be mutually reinforcing feedback loops, although the specific mechanisms and molecular components involved are still unclear. Taking into account the above, the purpose of this study is to determine whether Ca^2+^ signaling can regulate the transport of intestinal Gln in experimental animals and the underlying molecular mechanisms.

## Materials and Methods

### Animals

Harlan C-57BL/6 male mice were aged 6–8 weeks, weighing 21–23 g (purchased from Chongqing Tengxin Biotechnology Co., Ltd., Chongqing, China) were selected for the experiment. Mice were fed in a standard animal feeding room with an ambient temperature of 20–30°C, a humidity of 50–55%, a light/dark cycle for 12 h, and a free choice of food/water. All animal experiments complied with the requirements of the University’s Animal Ethics Committee and were approved by the University’s Animal Research Committee. The animals were randomly selected and aggregated in all experiments, and the data were collected and assessed blindly. The animals were killed when they sprained their necks under CO_2_ anesthesia.

### Tissue Preparation

After killing the animals as described above, the abdomen was opened layer by layer along the midabdominal line. In this study, the duodenum, proximal jejunum, or distal ileum mucosal tissues were mainly intercepted, with a length of about 5 cm. To ensure intestinal epithelial activity and avoid further damage, isolated intestinal segments were incubated in a 10 ml cold isotonic mannitol (300 mM) solution containing 10 μM indomethacin for 10 min. The intestinal tract was then opened longitudinally along with the mesentery. Food scraps were removed by scrubbing in the buffer, then dissected to remove the overlying smooth muscle and associated intermuscular nerves. Finally, segments were divided it into 3–4 chamber-sized tissues (window area, 0.1 cm^2^).

### Ussing Chamber Experiments

The treated mucosa of the small intestine was attached between chambers with an exposed area of 0.1 cm^2^. Experiments were performed under continuous short-circuited conditions (Voltage-Current Clamp, VCC MC6; Physiologic Instruments, San Diego, CA). After 15–30 min of basic parameters measurement, Gln was added to the mucosal side chamber of the tissue. When screening for molecular components, different drugs were added to the mucosal, serosal, or bilateral side for 10–20 min, followed by adding Gln. The transepithelial short-circuit currents (*I*_*sc*_) were measured *via* an automatic voltage clamp, in which μA was used for the original recordings, but μA/cm^2^ was used for summary data.

### Solution

The buffer solution on both sides of the Ussing chamber was prepared separately. The components of the mucosal solution (mM) were 115 NaCl, 1.2 MgCl_2_, 1.2 CaCl_2_, 25 sodium gluconate, 5.2 potassium gluconate, and 10 D-mannitol (final PH of 7.4) and gassed with 100% oxygen. Composition of serosal solution (mM): 115 NaCl, 1.2 MgCl_2_, 1.2 CaCl_2_, 25 NaHCO_3_, 2.2 K_2_HPO_4_, 0.8 KH_2_PO_4_, and 10 d-glucose (final PH of 7.4) in a carbon-oxygen mixture (5% CO_2_ and 95% O_2_, v/v). In the experiment, 3 ml of the above-mentioned solution were added to either side of the chamber at a constant temperature of 37°C. To create a low Na^+^ environment on the mucosal side, the Na^+^ in the mucosal solution was replaced with Li^+^ at the same concentration. For the low Ca^2+^ experiment, the concentration of CaCl_2_ was reduced to 0.5 mM; In the zero-Ca^2+^ experiment, Ca^2+^ in the solution was omitted, and EGTA (0.5 mM) was added to prevent potential Ca^2+^ contamination. Such solutions were isotonic for tissues.

### Materials

Sigma (Saint Louis, MO, United States) supplied l-Glutamine, spermine, cyclopiazonic acid (CPA), nifedipine, and gadolinium (III) chloride (GdCl_3_). GPNA hydrochloride, cinromide, Calhex 231 hydrochloride, NPS-2143, AMG 517, N, N, N′, N′- tetrakis (2-pyridylmethyl) ethylenediamine (TPEN), probenecid, SN-6, ouabain, mibefradil, and GSK-7975A were purchased from MedChemExpress (MCE; Monmouth Junction, NJ). Tocris Bioscience (Ellisville, MO) supplied 2-Aminoethyl diphenylborinate (2-APB), while APExBIO Technology LLC (Houston, TX) provided dantrolene. The other chemicals were obtained from BBI Life Science (Shanghai, China). Gln, Lithium chloride (LiCl), and Gadolinium (III) chloride (GdCl_3_) were dissolved in ultrapure water. Most of the remainder were prepared with dimethyl sulfoxide (DMSO), and the volume ratio was less than 1:1,000, which did not affect the basic current.

### Data and Statistical Analysis

The data and statistical analysis yield to the recommendations of Frontiers in Pharmacology. All the results were given as means ± SEM of the number of experimental tissues (n). The dose-response relationship of this study was obtained by nonlinear fitting analysis of Logistic curve parameters of experimental data. The net *I*_*sc*_ peak is the maximum peak stimulated by the drug minus the base level. Unpaired two-tailed *t*-test or one-way ANOVA was used, followed by Dunnett’s post-test to determine the statistical significance of the mean difference between the experimental groups. A follow-up analysis was performed when F reached *p* < 0.05 (GraphPad Prism 8.0), and there was no significant variance in inhomogeneity. Only a probability *p* < 0.05 is considered statistically significant.

## Result

### Gln is Mainly Transported in the Distal Ileum

**FIGURE 1:**
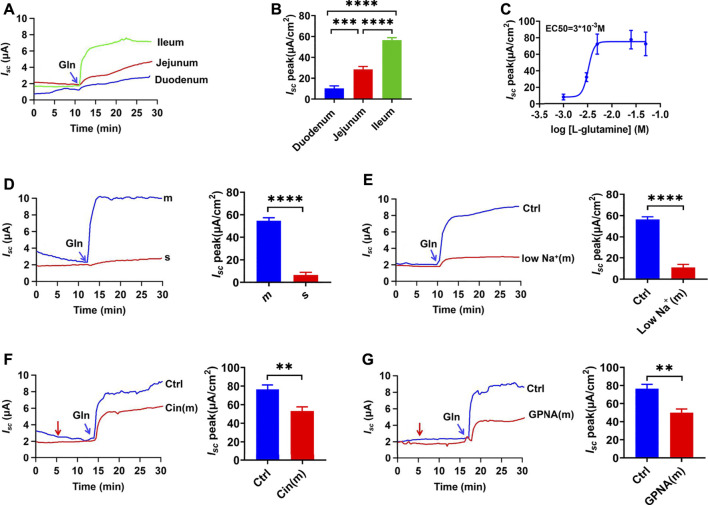
Luminal Gln is transported mainly in the ileum through NGcT. **(A)** Representative time courses of mucosal addition (indicated by the blue arrow) of Gln (25 mM)-stimulated *I*_*sc*_ of the duodenum, proximal jejunum, and distal ileum. **(B)** Comparisons of Gln-stimulated *I*_*sc*_ rising rates when added to the mucosal (m) side among different segments of the intestine (*n* = 6). **(C)** Representative examples of dose-response relations for adding Gln (1–50 mM) to the ileum mucosal side. (*n* ≥ 6). **(D)** Representative time courses of Gln (25 mM)-stimulated *I*_*sc*_ and the summary data of *I*_*sc*_ peak when added (indicated by the blue arrow) to the serosal (s) or mucosal (m) side of the ileum (*n* = 6). **(E)** Comparison of the distal ileum *I*_*sc*_ induced by mucosal side addition of Gln (25 mM) in mucosal normal Na^+^ condition (Ctrl, *n* ≥ 6) or the low-Na^+^ condition [low Na^+^ (m), Li^+^ replaced Na^+^,n = 6].**(F)** Representative time courses and summary data of Gln (5 mM)- stimulated *I*_*sc*_ peak in the absence (Ctrl, *n* ≥ 6) or the presence of cinromide (Cin(m), 25 μM, *n* = 6) added (indicated by the red arrow) to mucosal side of the ileum. **(G)** Representative time courses and summary data of Gln (5 mM) stimulated the *I*_*sc*_ peak in the absence (Ctrl, *n* ≥ 6) or the presence of GPNA (200 μM, *n* = 6) added (indicated by the red arrow) to mucosal side of the ileum. Results are presented as mean ± S.E. NS, no significant differences, **p < 0.01, ***p < 0.001, ****p < 0.0001 *vs.* corresponding control by one-way ANOVA followed Dunnett’s post-test.

We used the Ussing chamber experiment to determine if the entry of oral-Gln into the intestinal epithelium is an electrogenic process accompanied by ion transport. Firstly, we found the addition of Gln (25 mM) into the intestinal lumen induced short-circuit current (*I*_*sc*_), an instantaneous peak was followed by a continuous-time phase. Secondly, segmental differences in Gln-evoked *I*_*sc*_ in the mouse intestine were observed. As shown in Figure 1A, Gln induced much greater and faster *I*_*sc*_ in the distal ileum than in the proximal jejunum and the duodenum. As depicted in Figure 1B, Gln - *I*_*sc*_ increased from the proximal to the distal small intestine and mainly transported in the distal ileum. Finally, we analyzed the dose-response curve of ileal Gln-induced *I*_*sc*_. A maximum current (*I*_*sc*_ max) was 77.59 ± 4.25 μA/cm^2^ and an EC50 was 3 x 10^−3^ M (Figure 1C).

### Gln Induces Ileal *I*_*sc*_ *via* the NGcT From the Luminal Side

There are different Gln transporters on IEC’s apical and basolateral sides, so we tested which side was involved in Gln transport in the distal ileum. The mucosal application of Gln induced a significant *I*_*sc*_, but the serosal application could not induce any detectable *I*_*sc*_ (Figure 1D), clearly indicating luminal Gln transport. Then reducing the concentration of Na^+^ in mucosal solution significantly reduced the Gln-induced *I*_*sc*_ compared to the control group, indicating that intestinal Gln transport requires Na^+^ to be accompanied, namely the luminal Na^+^/Gln co-transporters (NGcT) (Figure 1E). Finally, either cinromide (25 µM), a selective inhibitor of epithelial NGcT (B^0^AT1) (Danthi et al., 2019), or GPNA (200 µM), a non-specific inhibitor of NGcT (Broer et al., 2016), attenuated Gln (5 mM)-stimulated *I*_*sc*_ peak after it was added to the mucosal side (Figures 1F,G).

### The CaSR Activation is not Involved in the Ileal Na^+^/Gln Co-Transport

**FIGURE 2:**
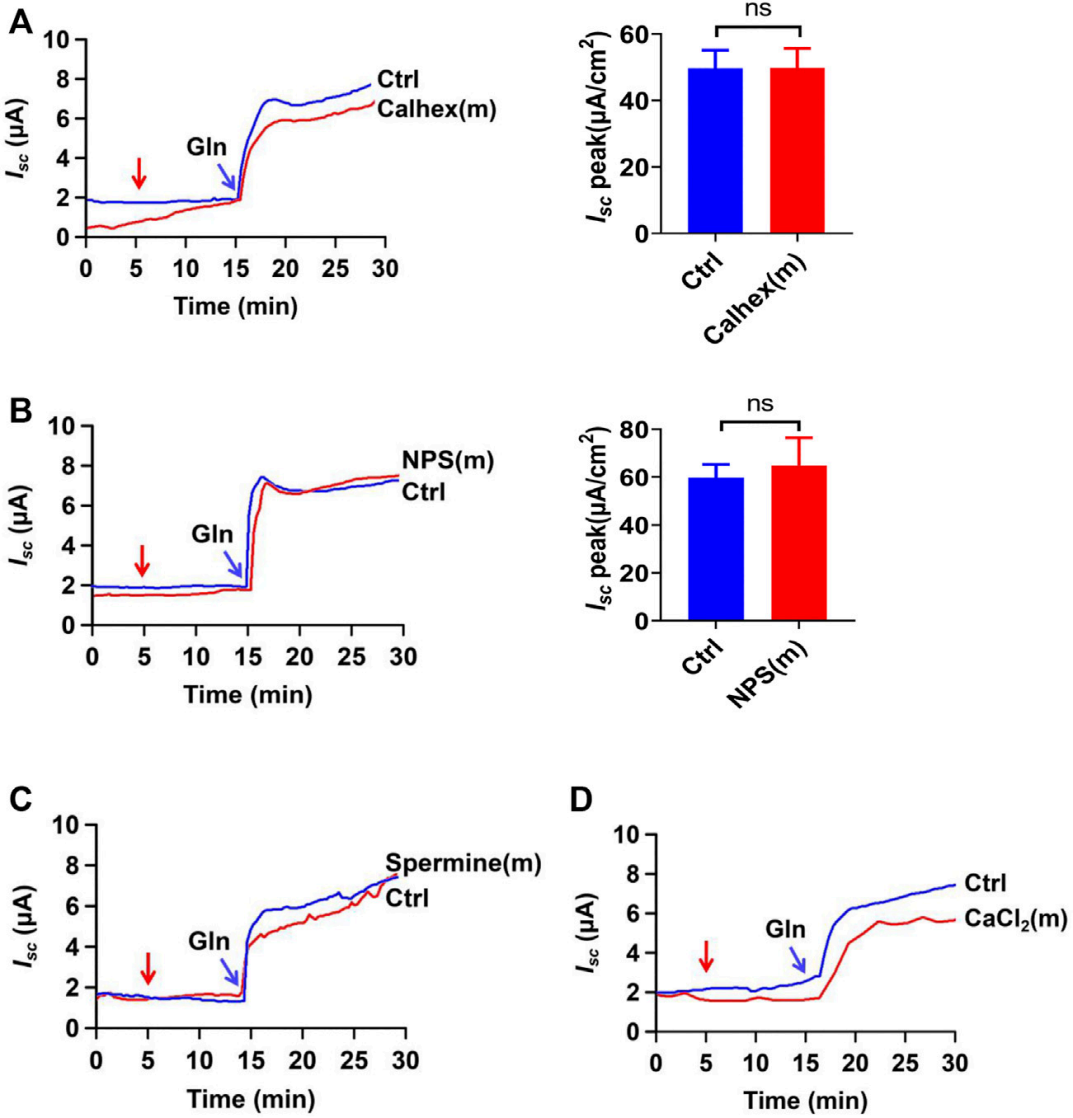
Gln-induced ileal *I*_*sc*_ is not mediated by CaSR activation. **(A)** Representative time courses and summary data showing the effect of mucosal (m) addition of Calhex 231 (Calhex, 10 μM, *n* = 5) on Gln (5 mM)-stimulated *I*_*sc*_. The red arrow indicates the time of drug addition. Ctrl (*n* ≥ 6) represents the control without drug treatment. **(B)** Representative time courses and summary data of Gln (25 mM)-stimulated *I*_*sc*_ after mucosal addition of NPS-2143 (NPS, 30 μM, *n* = 5). The red arrow indicates the time of drug addition. Ctrl (*n* ≥ 6) is the control group without drug treatment. **(C)** Representative time courses showing the effect of mucosal addition of spermine (3 mM) (indicated by the red arrow) on Gln (5 mM)-stimulated *I*_*sc*_. **(D)** Representative time courses showing the effect of mucosal addition of CaCl_2_ (5 mM) (indicated by the red arrow) on Gln-stimulated *I*_*sc*_. Results are presented as mean ± S.E. NS, no significant differences vs. corresponding control by Student’s unpaired, two-tailed *t*-test.

In addition to the Na^+^ -coupled Gln transporters, the calcium-sensitive receptors (CaSR) may be activated by Gln as an L-amino acid to participate in intestinal Gln response *via* Gq/Ca^2+^ signaling pathway (Joshi et al., 2013). To examine whether the CaSR activation is involved in ileal Gln-induced *I*_*sc*_, calhex 231 (10 µM) and NPS-2143 (30 µM), two selective CaSR antagonists, were first applied; but neither of them altered the ileal Gln-induced *I*_*sc*_ (Figures 2A,B). Secondly, spermine (3 mM) and CaCl_2_ (5 mM), two CaSR activators, did not induce any currents by themselves; nor did they affect the Gln-induced ileal *I*_*sc*_ (Figures 2C,D). These data clearly exclude the involvement of the CaSR in the Gln-induced ileal *I_sc_* or Na^+^/Gln co-transport.

### Luminal Ca^2+^ is Required for Ileal NGcT

**FIGURE 3:**
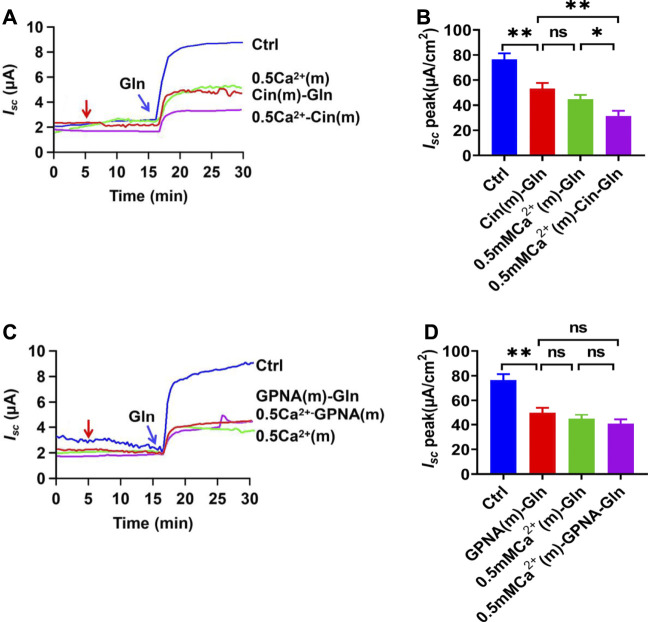
Gln ileal transport is luminal Ca^2+^-dependent. **(A)** Representative time courses showing the effect of reduced Ca^2+^ concentration in mucosal side [0.5 mM Ca^2+^ (m)-Gln] and the addition of cinromide (add at the red arrow) on Gln (5 mM)-stimulated *I*_*sc*_. Ctrl represents Gln-induced *I*_*sc*_ at the normal Ca^2+^ concentration (1.2 mM Ca^2+^) on the mucosal side without any treatment. **(B)** Summary data of Gln-stimulated *I*_*sc*_ after mucosal addition of single 25 µM Cinromide [Cin (m)-Gln, *n* = 7], single reduced mucosal Ca^2+^ [0.5 Ca^2+^ (m)-Gln, *n* = 6], or both of them [0.5 mM Ca^2+^ (m) -Cin-Gln, *n* = 6]. Compared with Gln (Ctrl, 5 mM, *n* ≥ 6) induced *I*_*sc*_ without any treatment. **(C)** Representative time courses of Gln (5 mM) -stimulated *I*_*sc*_ after Ca^2+^ reduction [0.5 Ca^2+^(m)], and addition of GPNA (add at the red arrow) to normal Ca^2+^ or 0.5 mM Ca^2+^ solutions. Ctrl represents the control without any treatment. **(D)** Summary data of Gln-stimulated *I*_*sc*_ after mucosal addition of single GPNA [GPNA (m)-Gln, *n* = 6], single reduced mucosal Ca^2+^ [0.5 mM Ca^2+^ (m), *n* = 6], or the presence of GPNA (500 µM) in lower Ca^2+^ condition [0.5 mM Ca^2+^ (m)-GPNA-Gln, *n* = 6]. Compared with Gln-induced *I*_*sc*_ without any treatment. Results are presented as mean ± S.E. NS, no significant differences, *p < 0.05, **p < 0.01, ***p < 0.001 *vs.* corresponding control by Student’s unpaired, one-way ANOVA followed Dunnett’s post-test.

To investigate whether luminal Ca^2+^ is important in Na^+^/Gln co-transport, extracellular Ca^2+^ on the mucosal side was reduced to a low Ca^2+^ concentration (0.5 mM). As shown in Figures 3A,B, Gln-induced *I*_*sc*_ was significantly reduced in low Ca^2+^ concentration, suggesting its important role. Interestingly, luminal addition of cinromide further reduced Gln-induced *I*_*sc*_ in low Ca^2+^ concentration (Figures 3A,B), but GPNA did not (Figures 3C,D), suggesting that the sensitivities of NGcT to luminal Ca^2+^ may be different.

**FIGURE 4:**
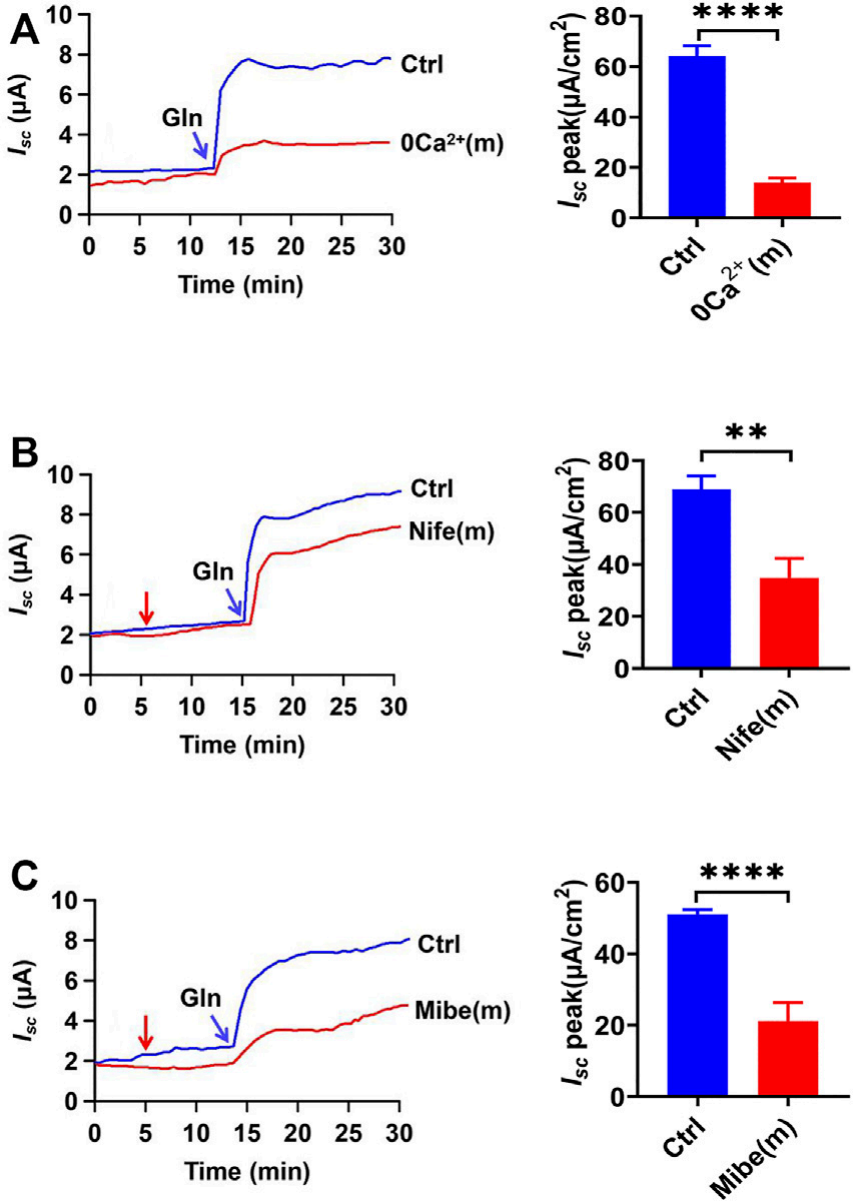
Gln-*I*_*sc*_ is luminal VGCC-dependent. **(A)** Representative time courses and summary data of Gln (25 mM) -stimulated *I*_*sc*_ after mucosal Ca^2+^ omission [0 Ca^2+^ (m), *n* = 6]. Ctrl (*n* ≥ 6) represents the control in which normal Ca^2+^ (1.2 mM Ca^2+^) was in both mucosal and serosal sides. **(B)** Representative time courses of Gln (25 mM)-stimulated *I*_*sc*_ and the summary data of *I*_*sc*_ peak in the absence (Ctrl) or the presence of nifedipine [Nife (m), 10 μM, *n* = 6] added (indicated by the red arrow) to mucosal side of the ileum. **(C)** Representative time courses and summary data of Gln (5 mM) -stimulated ileal *I*_*sc*_ peak after mucosal side addition of mibefradil [Mibe (m), 30 μM, *n* = 6]. The red arrow indicates the time of inhibitor addition. Ctrl (*n* ≥ 6) represents the control without drug treatment. Results are presented as mean ± S.E. NS, no significant differences, **p < 0.01, ****p < 0.0001 *vs.* corresponding control by Student’s unpaired, two-tailed *t*-test.

To further study the role of Ca^2+^ in Na^+^/Gln co-transport, luminal Ca^2+^ was omitted. Ca^2+^ omission almost abolished Gln-induced *I*_*sc*_ (Figure 4A), indicating that luminal Ca^2+^ is not only vital in ileal Gln electrogenic transport but also dose-dependently regulates this process.

### Role of Luminal Ca^2+^ Entry *via* the VGCC in Ileal Na^+^/Gln Co-Transport

To elucidate the mechanisms of how luminal Ca^2+^ regulates ileal Gln electrogenic transport, we analyzed the role of Ca^2+^ channels in the regulation of Gln -induced *I*_*sc*_. Luminal addition of nifedipine (10 µM), a selective blocker of the L-type voltage-gated Ca^2+^ channels (VGCC), significantly inhibited Gln-induced *I*_*sc*_ (Figure 4B). In addition, mibefradil (30 µM), a selective blocker of the T-type VGCC (Mehrke et al., 1994), also inhibited Gln-induced ileal *I*_*sc*_ after added to the luminal side (Figure 4C). Therefore, luminal Ca^2+^ influx *via* VGCC is critical for ileal Na^+^/Gln co-transport.

### Role of Serosal Ca^2+^ Entry *via* TRPV Channels in Gln-*I*_*sc*_

**FIGURE 5:**
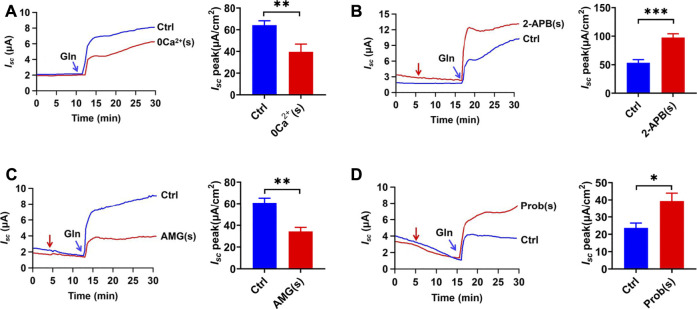
Gln-*I*_*sc*_ is basolateral Ca^2+^ -dependent. **(A)** Comparison of ileal *I*_*sc*_ induced by mucosal side addition of 25 mM Gln in the presence (Ctrl, *n* = 6) or the absence of serosal Ca^2+^ [0 Ca^2+^ (s), *n* = 6]. **(B)** Representative time courses and summary data of Gln (25 mM) -stimulated *I*_*sc*_ peak after serosal addition of 2-APB [2-APB (s), 100 μM, *n* = 6]. The red arrow indicates the time of drug addition. Ctrl represents the control without any treatment. **(C)** Representative time courses and summary data showing the effects of serosal addition of AMG 517 [AMG (s), 100 μM, *n* = 6] on Gln (5 mM)-induced ileal *I*_*sc*_ peak. The red arrow indicates the time of drug addition. Compared with a control group (Ctrl, *n* ≥ 6) without any drug treatment. **(D)** Representative time courses and summary data of Gln (3 mM) -stimulated *I*_*sc*_ peak after serosal addition of probenecid [Prob (s), 100 μM, *n* = 5]. The red arrow indicates the time of drug addition. Ctrl (*n* ≥ 6) represents the control without any treatment. Results are presented as mean ± S.E. NS, no significant differences, *p < 0.05, **p < 0.01, ***p < 0.001 *vs.* corresponding control by Student’s unpaired, two-tailed *t*-test.

To examine whether serosal Ca^2+^ also participates in Gln ileal response, we investigated the Gln-evoked *I*_*sc*_ after omitting serosal Ca^2+^. As shown in Figure 5A, serosal Ca^2+^ omission markedly inhibited Gln-evoked *I*_*sc*_, indicating the importance of serosal Ca^2+^.

Since TRPV as Ca^2+^-permeable channels are expressed and function in the ileum (Allais et al., 2020; Manneck et al., 2021), we tested whether Ca^2+^ influx *via* the TRPV channels on the serosal side regulates Na^+^/Gln co-transport. Firstly, serosal application of 2-Aminoethyl diphenylborinate (2-APB) (100 µM) (Hu et al., 2004), an activator of TRPV1/2/3 channels, significantly enhanced Gln-induced ileal *I*_*sc*_ (Figure 5B). Secondly, serosal application of AMG 517 (100 µM), a selective blocker of TRPV1 channels, significantly attenuated ileal Gln-*I*_*sc*_ (Figure 5C). Finally, probenecid (100 µM), a selective activator of TRPV2 channels, enhanced Gln-induced *I*_*sc*_ (Figure 5D). Therefore, serosal Ca^2+^ entry *via* TRPV1/2 channels plays a role in ileal Gln-*I*_*sc*_.

### The Serosal SOCE Mechanism in Ca^2+^-Mediated Gln-*I*_*sc*_

**FIGURE 6:**
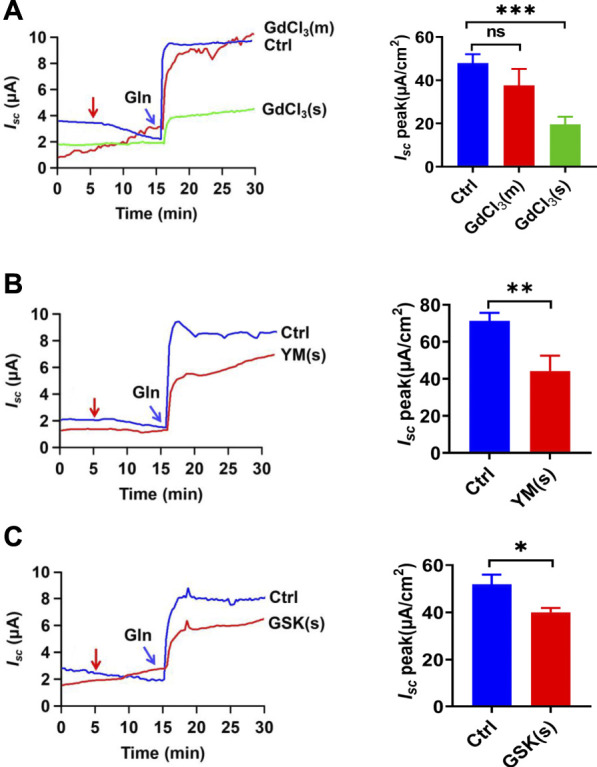
CRAC channels in the regulation of Gln-stimulated ileal Isc. **(A)** Representative time courses of Gln-stimulated *I*_*sc*_ and the summary data of *I*_*sc*_ peak in the absence (Ctrl) or the presence of GdCl_3_ (30 μM, *n* = 6), added (indicated by the red arrow) to mucosal (m) or serosal (s) side of the ileum. **(B)** Representative time courses and summary data of the effect of serosal addition of YM-58483 [YM (s), 0.3 μM, *n* = 6] on Gln-stimulated ileal *I*_*sc*_ peak. Compared with a control group (Ctrl, *n* ≥ 6) without any drug treatment. **(C)** Representative of the time courses and summary of Gln-stimulated ileal *I*_*sc*_ after the serosal addition of GSK-7975A [GSK (s), 100 μM, *n* = 6]. The red arrow indicates the time of drug addition. Ctrl (*n* ≥ 6) represents the control without any treatment. Results are presented as mean ± S.E. NS, no significant differences, *p < 0.05, **p < 0.01, ***p < 0.001 *vs.* corresponding control by Student’s unpaired, two-tailed *t*-test or one-way ANOVA followed Dunnett’s post-test.

Since the store-operated Ca^2+^ entry (SOCE) is a well-established mechanism to regulate the Ca^2+^-dependent functions in IEC, including jejunal glucose absorption (Zhang et al., 2021),we examined whether the SOCE mechanism regulates ileal Gln-*I*_*sc*_ and started with GdCl_3_, a commonly used SOCE blocker (Zhu et al., 2020). As shown in Figure 6A, serosal addition but not mucosal addition of GdCl_3_ (30 µM) inhibited Gln-induced *I*_*sc*_, consistently with the previous finding of the serosal localization of the SOCE (Zhang et al., 2021). Secondly, serosal application of YM-58483 (0.3 µM) and GSK-7975A (100 µM), two selective SOCE blockers with different structures, also reduced Gln-*I*_*sc*_ (Figures 6B,C). Thus, the serosal SOCE mechanism is involved in the Ca^2+^-dependent ileal Gln-*I*_*sc*_.

### Role of the ER Ca^2+^ Store in Ileal Na^+^/Gln Co-Transport

**FIGURE 7:**
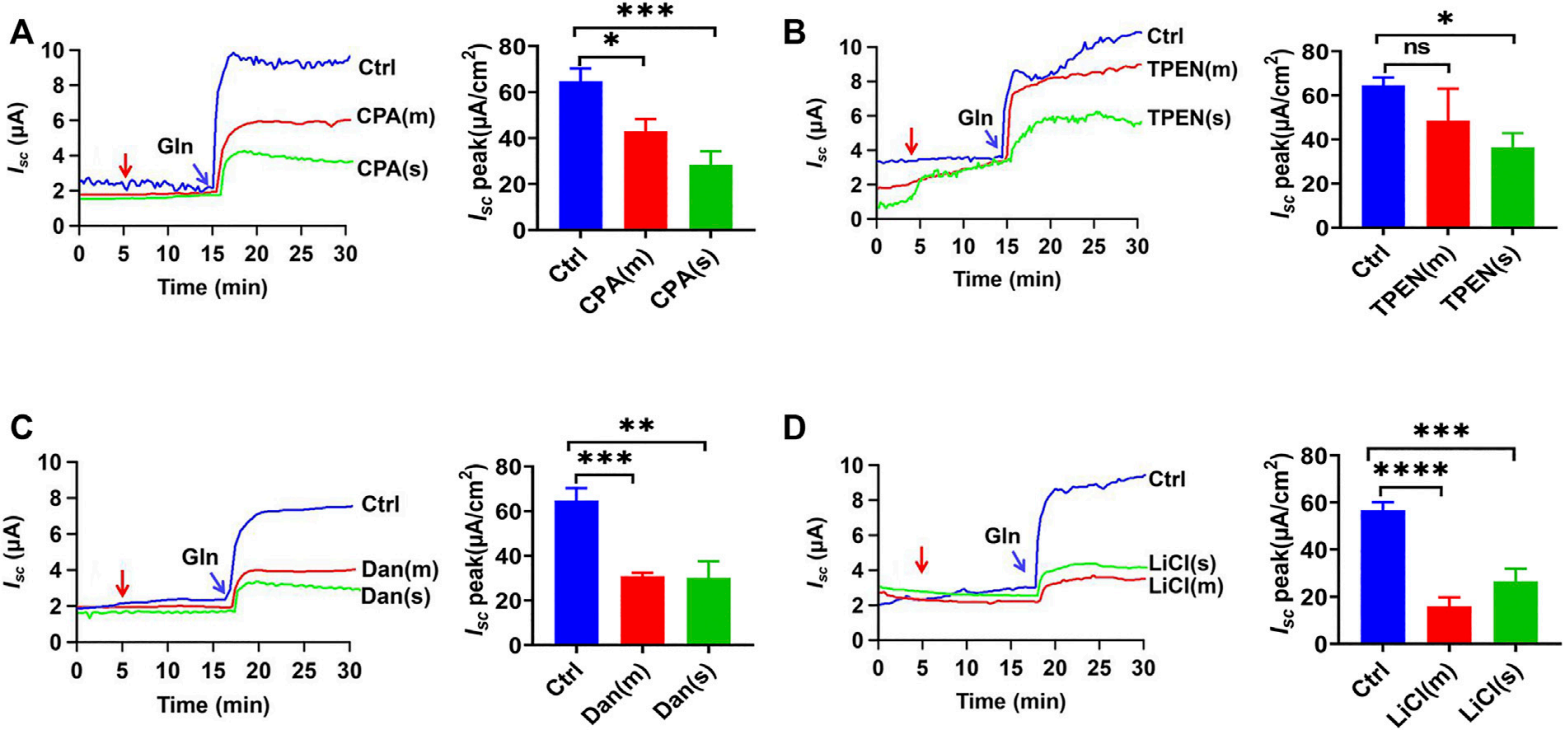
Roles of ER Ca^2+^ storage in ileal Gln transport. **(A)** Representative time courses and summary data showing the effect of mucosal (m) or serosal (s) addition of CPA (30 μM, *n* = 6 for each) on Gln (5 mM)-stimulated *I*_*sc*_. The red arrow indicates the time of drug addition. Ctrl (*n* ≥ 6) represents the control without drug treatment. **(B)** Representative time courses and summary data of Gln-stimulated *I*_*sc*_ after mucosal (m) or serosal (s) addition of TPEN (1 mM, *n* = 6 for each). The red arrow indicates the time of drug addition. Ctrl (*n* ≥ 6) is the control group without drug treatment. **(C)** Representative time courses and summary data showing the effect of mucosal (m) or serosal (s) addition of dantrolene (Dan, 100 μM, *n* = 6) (indicated by the red arrow) on Gln (5 mM)-stimulated *I*_*sc*_. Compared with a control group (Ctrl *n* ≥ 6) without any drug treatment. **(D)** Representative time courses and summary data showing the effect of mucosal (m) or serosal (s) addition of LiCl (30 mM, *n* = 6) (indicated by the red arrow) on Gln-stimulated *I*_*sc*_. Compared with a control group (Ctrl *n* ≥ 6) without any drug treatment. Results are presented as mean ± S.E. NS, no significant differences, *p < 0.05, **p < 0.01, ***p < 0.001, ****p < 0.0001 *vs.* corresponding control by Student’s unpaired, one-way ANOVA followed Dunnett’s post-test.

We further explored the role of intracellular ER Ca^2+^ in Gln-*I*_*sc*_. Firstly, cyclopiazonic acid (CPA), an inhibitor of endoplasmic reticulum calcium ATPase (ERCA), can indirectly deplete the ER Ca^2+^. Either mucosal or serosal application of CPA (30 µM) significantly reduced Gln-stimulated *I*_*sc*_ (Figure 7A). Secondly, we used N, N, N′, N′-tetrakis (2-pyridylmethyl) ethylenediamine (TPEN), a membrane-permeable ER calcium chelator. TPEN (1 mM) inhibited Gln-*I*_*sc*_ (Figure 7B). Therefore, The ER Ca^2+^ is required for ileal Na^+^/Gln co-transport.

Thirdly, to further explore whether the ER Ca^2+^ release through IP_3_R and RyR participate in Gln-*I*_*sc*_, we applied the selective RyR inhibitor dantrolene (300 µM), which significantly inhibited Gln-induced *I*_*sc*_ on each side of the ileum (Figure 7C). Moreover, LiCl (30 mM) was used to inhibit IP_3_ production, and its addition to each side also obviously attenuated Gln-induced *I*_*sc*_ (Figure 7D). Taken together, the ER Ca^2+^ release *via* IP_3_R and RyR is required for ileal Na^+^/Gln co-transport.

### The Role of Serosal NKA and NCX in Ileal Gln-*I*_*sc*_

**FIGURE 8:**
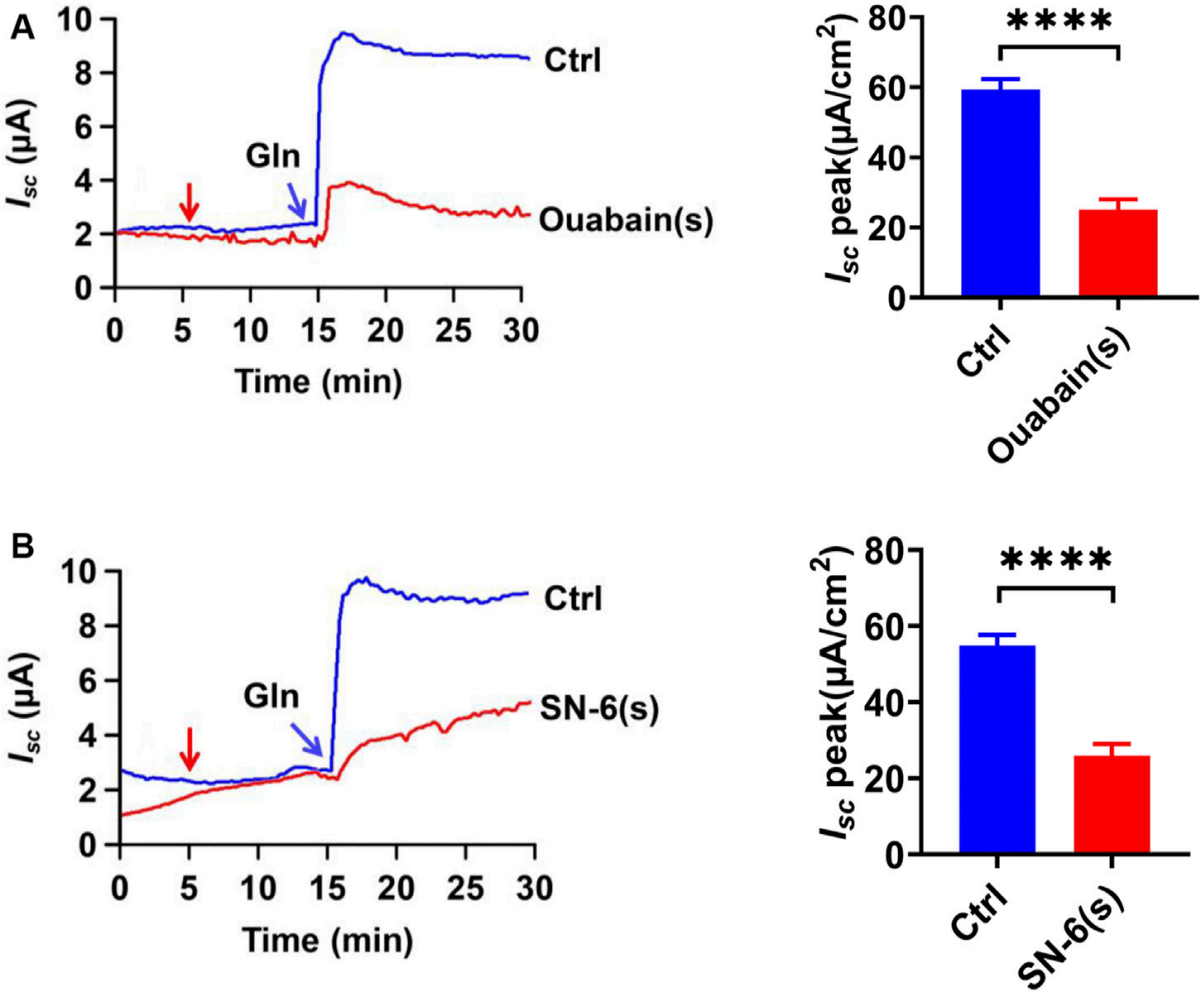
The NKA and NCX in Ca^2+^-mediated Gln transport. **(A)** Representative time courses and summary data showing the effect of serosal (s) addition of ouabain [Ouabain (s), 1 mM, *n* = 6] (indicated by the red arrow) on Gln-induced *I*_*sc*_, compared to a control group (Ctrl, *n* ≥ 6) without any drug treatment. **(B)** Representative time courses and summary data showing the effect of serosal addition of SN-6 [SN-6 (s), 10 μM, *n* = 6] on Gln-stimulated *I*_*sc*_ (indicated by the red arrow). Ctrl (*n* ≥ 6) is the control group without drug treatment. Results are presented as mean ± S.E. NS, no significant differences, ****p < 0.0001 *vs.* corresponding control by Student’s unpaired, two-tailed *t*-test.

Since Na^+^-coupled solute transport across the epithelium requires serosal Na^+^/K^+^ ATPase (NKA) to maintain the transmembrane potential of Na^+^, we tested whether it is involved in ileal Gln-*I*_*sc*_. Serosal addition of ouabain (1 mM), a selective inhibitor of NKA, significantly attenuated Gln-induced *I*_*sc*_ (Figure 8A). We also tested the involvement of the serosal Na^+^/Ca^2+^ exchanger (NCX), a bidirectional transporter of Na^+^ and Ca^2+^. The serosal addition of SN-6 (10 µM), a selective NCX inhibitor, significantly reduced Gln-induced *I*_*sc*_ (Figure 8B). Therefore, serosal NKA and NCX are involved in ileal Gln-*I*_*sc*_.

## Discussion

As Ussing Chamber is widely used to study ion, drug, and nutrient transport in intestinal epithelium (He et al., 2013; Hempstock et al., 2021; Michiba et al., 2021), and we applied it to investigate the regulation of Gln transport. In our study the Gln-induced ileal *I*_*sc*_ well represents the Na^+^/Gln co-transport since, 1) the mucosal application of Gln induced a significant *I*_*sc*_, but the serosal application could not induce any detectable *I*_*sc*_; 2) lowering the concentration of Na^+^ in mucosal solution significantly reduced the Gln-induced *I*_*sc*_; 3) either cinromide, a selective inhibitor of epithelial NGcT (B^0^AT1), or GPNA, a non-specific inhibitor of NGcT, attenuated Gln-stimulated *I*_*sc*_ peak after added to the mucosal side. This notion has been well proved by several previous reports (Rexhepaj et al., 2006; Rexhepaj et al., 2007; Ducroc et al., 2010). Unlike glucose transports, Gln has a diverse and non-selective transport systems. According to expression, distribution and functional specificity of intestinal Gln transporters, our data suggest that NGcT (B^0^AT1) may play a major role in this process, but the other transporters cannot be ruled out completely (Broer and Fairweather 2019; Chen et al., 2020).

Gln has been widely applied for a long time in clinical practice due to its multiple biological actions in humans. Since most of the Gln in the enteral diet is oxidized and metabolized in the GI tract to produce ATP, it is so-called “intestinal fuel” (Curi et al., 2005). Gln is also an important nitrogen source for synthesizing nucleic acids and proteins that inhibit cell apoptosis and enhance intestinal cell proliferation. Moreover, Gln affects tight junction protein expression and intestinal barrier integrity through Ca^2+^/CaMKK2-AMPK signaling (Wang et al., 2016). In addition to its physiological role, Gln and its transporters are also potential targets for various diseases, such as intestinal inflammation, cancer, and metabolic diseases (Avissar et al., 2004; Javed et al., 2018; Ye et al., 2018; Yadav et al., 2020). Therefore, it is important to study the exact regulatory mechanisms of intestinal Gln transport.

Although the important role of Gln in nutrition and function of the GI tract is well known (Xue et al., 2011; Li et al., 2019), the exact regulatory mechanisms of its transport in the ileum are not fully understood. Particularly, it has not been explored if Ca^2+^ signaling plays a role in this regulation. In the present study, we demonstrate for the first time that, 1) [Ca^2+^]_i_ in IEC plays an essential role in the regulation of ileal Gln-*I*_*sc*_ without the involvement of the CaSR activation by Gln; 2) luminal Ca^2+^ entry through apical VGCC to regulate ileal Na^+^/Gln co-transport; 3) serosal Ca^2+^ entry through TRPV1/2, SOC channels, and NCX to mediate ileal Na^+^/Gln co-transport; 4) the ER Ca^2+^ release *via* the RyR and IP_3_R may also trigger ileal Na^+^/Gln co-transport. Therefore, our findings not only reveal the important role of [Ca^2+^]_i_ in the regulation of ileal Gln transport but also provide new insights into the mechanisms of Ca^2+^-mediated nutrient absorption in the ileum.

Most studies on intestinal nutrient absorption (such as dipeptide and glucose) focus on the proximal small intestine (such as the duodenum and the jejunum) but not on the distal small intestine (such as the ileum). However, the ileum is the longest segment of the intestine, where the chyme has the longest residence time to complete nutrient absorption. Our study also showed that Gln induced faster and larger *I*_*sc*_ in the ileum than in the proximal intestine.

As a well-known second messenger, [Ca^2+^]_i_ regulates many physiological functions in mammalian cells, such as neural activity, hormone secretion, muscle cell contraction, etc. Yet, studies on Ca^2+^ regulation of intestinal nutrient absorption are rare, particularly, the survey on Ca^2+^ regulation of ileal Gln transport is still lacking. The ileum is involved in the active Ca^2+^ transport when luminal Na^+^ coupled-nutrients are high (Kellett 2011), and it may play a more important role than the duodenum in adapting to hypocalcemia (Dhawan et al., 2017). Therefore, it is reasonable to speculate that Ca^2+^ and Na^+^/Gln co-transport might interact in the ileum. In the present study, we are the first to apply the native ileum of mice to investigate the Ca^2+^ regulation of ileal Gln transport and found an essential role of Ca^2+^ for this regulation.

There are two primary sources of Ca^2+^ in the cytoplasm in IEC: extracellular Ca^2+^ entry *via* plasma membrane and intracellular Ca^2+^ release from the endoplasmic reticulum (ER). The VGCC has been reported to express on the luminal side of jejunal and ileal epithelium in mice (Reyes-Fernandez and Fleet 2015; Beggs et al., 2019). We reveal that Ca^2+^ entry through luminal VGCC plays a key role in Gln transport, consistent with our previous jejunal glucose absorption report. Like in the process of jejunal Na^+^/glucose co-transporter, ileal Gln transport *via* Na^+^/Gln co-transporter also depolarizes plasma membrane to induce Ca^2+^ entry through luminal VGCC (Kellett 2011; Nakamura et al., 2020). The Ca^2+^ entry may activate inositol 1,4,5-triphosphate receptor (IP_3_R) and ryanodine receptor (RyR) to trigger the ER Ca^2+^ release (so-called CICR) (Rahman 2012; Zhang et al., 2021). Indeed, ileal Gln-*I*_*sc*_ is inhibited by either the ER Ca^2+^ depletion with CPA and TPEN or inhibition of IP_3_ production and selective blockade of RyR. ER Ca^2+^ depletion leads to extracellular Ca^2+^ entry, known as the SOCE mechanism, which plays a key role in regulating Ca^2+^-dependent biological functions in IEC, such as in jejunal glucose absorption (Zhang et al., 2021) and ileal Gln transport here.

Moreover, several ion channels and transporters express and function on the basolateral side of the small intestinal epithelium (Dong et al., 2005; Tsuchiya et al., 2014; Manneck et al., 2021, Yinghui Cui1 2021). Recently, the expression and function of transient receptor potential (TRP) channels in the GI tract have attracted extensive attention; TRPV1-6 channels have been shown to be stably expressed in IEC as non-selective cation channels with Na^+^ and Ca^2+^ permeability (Voets et al., 2004; Rao et al., 2006; Ueda et al., 2009; Fothergill et al., 2016; Maurino et al., 2020; Manneck et al., 2021). Although TRPV channels in GI sensory neurons have been studied extensively (De Petrocellis et al., 2012; Maurino et al., 2020; Grover et al., 2021), our study is the first to demonstrate the involvement of Ca^2+^ entry *via* TRPV channels in the regulation of ileal Gln transport in IEC. Na^+^/Ca^2+^ influx mediated by TRPV channels could activate bidirectional ion exchange (NCX), whose transport direction depends on Na^+^ and Ca^2+^ gradients and membrane potentials (Zhang J. et al., 2019; Zhao et al., 2021). Furthermore, we found that NKA on the serosal side of the intestine are also involved in ileal Gln-*I*_*sc*_, although it is well known that NKA activity is essential to provide potential energy for epithelial ion transports. Therefore, we have identified two Ca^2+^-permeable channels and two ion transporters on the serosal side of the ileum, which likely work together to regulate intestinal Ca^2+^-dependent Gln-*I*_*sc*_. [Ca^2+^]_i_ has a variety of possibilities in regulating Gln transport solely or synergistically: on the one hand, studies have shown that the Ca^2+^-activated PKC directly regulates Gln transporters (Dong et al., 2018). On the other hand, Ca^2+^ has been shown to stimulate NKA or K^+^ channels to maintain potential differences across apical membranes for Na^+^-coupled nutrient transport (Nepal et al., 2021).

**FIGURE 9:**
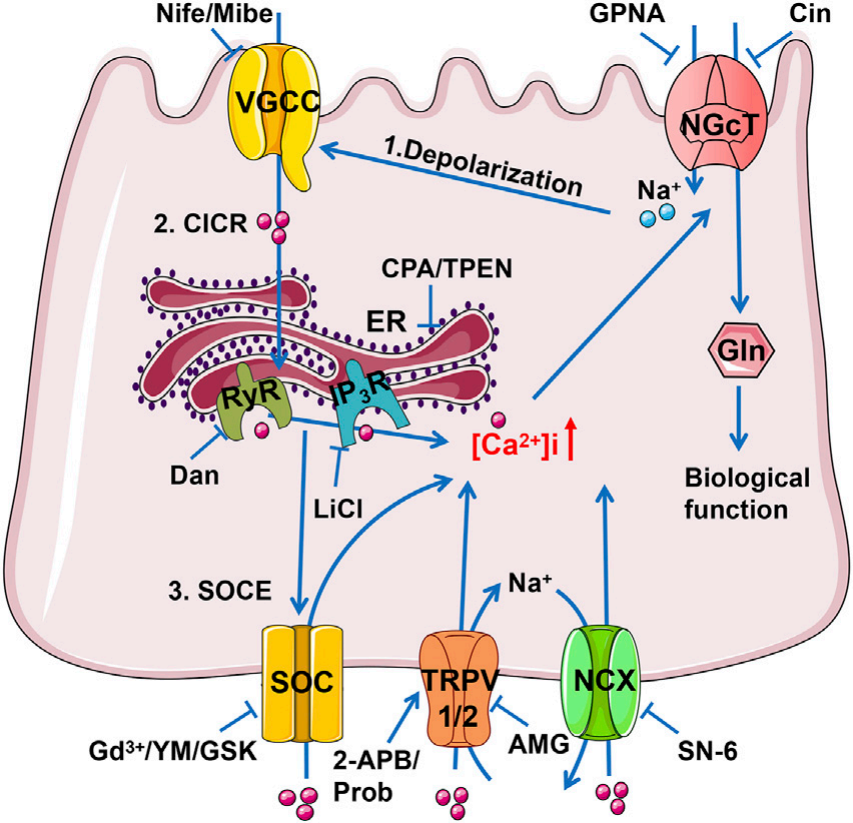
The schematic diagram depicts the mutual achievement of Ca^2+^ and Gln transport in the ileum. Gln entry through electrogenic Na^+^/Gln co-transporter causes apical membrane depolarization, which activates VGCC causing Ca^2+^ influx. Ca^2+^ flowing from VGCC can activate IP_3_R and RyR mediated ER Ca^2+^ release (CICR), and the depleted Ca^2+^ storage may activate SOC channels to complete the SOCE mechanism of more Ca^2+^ inflow. The raised [Ca^2+^]_i_ activates TRPV1/2 channels, leading to not only Ca^2+^ entry but Na^+^ entry as well. Na^+^ entry would reverse NCX to further enhance Ca^2+^ entry. Finally, the raised [Ca^2+^]_i_ promotes ileal Na^+^ and Gln entry *via* NGcT. Gln, Glutamine; NGcT, Na^+^/Gln co-transporters; ER, endoplasmic reticulum; RyR, ryanodine receptor; IP_3_R, inositol 1,4,5-triphosphate receptor; SOC, store-operated Ca^2+^ channel; TRPV, transient receptor potential vanilloid channel; NCX, Na^+^/Ca^2+^ exchanger.

Using native intestinal tissues we have provided functional evidence that the transport of dietary Gln and Ca^2+^ is mutually regulated in the ileum. Figure 9 depicts the proposed mechanisms of this mutual regulation: 1) the Na^+^ entry *via* electrogenic Na^+^-Gln co-transporter depolarizes the apical membrane to cause Ca^2+^ entry *via* VGCC in IEC, triggering the CICR *via* IP_3_R and RyR; 2) the ER/Ca^2+^ release induces the SOCE mechanism to activate TRPV1/2 channels, leading to not only Ca^2+^ entry but Na^+^ entry; 3) Na^+^ entry would reverse NCX to further enhance Ca^2+^ entry; 4) the raised [Ca^2+^]_i_ finally promotes ileal Gln entry *via* Na^+^-Gln co-transporter. There may be an alternative explanation that calcium channels are regulated by Gln *via* the CaSR, however, our data have clearly excluded this alternative explanation, further supporting the notion described above that is consistent with a recent report in IEC (Nakamura et al., 2020).

In conclusion, we uncovered a novel Ca^2+^ regulatory mechanism of ileal Gln transport. Since Gln is intestinal fuel and has multiple biological functions in the GI tract, the Ca^2+^ regulation of ileal Gln transport may be significant in GI health and disease. The combining application of Ca^2+^ and Gln in enteral and parenteral solutions or targeting the corresponding channels may have therapeutic potentials to improve GI nutrition and function.

## Data Availability

The original contributions presented in the study are included in the article/Supplementary Material, further inquiries can be directed to the corresponding authors.
